# The influence of uncertain expectations on Chinese rural residents’ consumer behavior decisions: Theoretical analysis and empirical test

**DOI:** 10.3389/fpsyg.2022.1052962

**Published:** 2023-01-05

**Authors:** Lina Han, Xiaofei Liu, Shusheng Ma

**Affiliations:** ^1^School of Public Finance & Taxation, Guangdong University of Finance and Economics, Guangzhou, China; ^2^Postdoctoral Programme of China Centre for Industrial Security Research, Beijing Jiaotong University, Beijing, China; ^3^Beijing Laboratory of National Economic Security Early-warning Engineering, Beijing Jiaotong University, Beijing, China; ^4^Jilin Business and Technology College, Changchun, China

**Keywords:** uncertain expectations, consumption behavior decision, precautionary saving, rural residents, empirical analysis

## Abstract

Economic uncertainty will result in consumers’ unstable environment expectations, thus affecting household consumption decisions. The paper established a consumption model though using the cointegration theory and ECM model, and used the annual data from 2002 to 2021 to examine the influence of uncertain expectations on the consumption behavior of rural residents in China. The research results show that consumers’ income, consumption, consumption habits liquidity constraints and precautionary savings jointly produce the feelings of uncertainty, which will lead to rural consumers’ uncertain expectations. Uncertain expectations will not only lead to rural residents the prudent consumption behavior of residents, but also lead to the lack of confidence of residents, forming phased consumption characteristics. Combining the results of the empirical analysis, this paper gives suggestions to promote rural consumption from the perspective of increasing rural residents’ income, reforming the income distribution system, improving the rural financial system, and reinforcing the construction of rural consumption infrastructure.

## Introduction

The world is now undergoing major changes. As the pandemic continuously impedes global economic development, the global economy is faced with great uncertainties. Economic uncertainties will result in consumers’ unstable environment expectations, thus affecting enterprises’ investment and financing decisions and household consumption decisions. Uncertainties constitute ineluctable objective reality during economic development. Normally, certain economic variables lead to uncertainties; and necessary connections can be seen between the current changes in external environment and uncertainties. Consumer behaviors are affected by uncertainties, as consumers with uncertain expectations are more irrational. Such irrational expectations will not only lead to residents’ caution about consumption, motivate their precautionary savings, thus reduce their consumption, but also undermine their confidence so that risk-averse rational consumers attempt to seek “certainties” in an uncertain environment. On this basis, this paper will explain how uncertainties induce residents’ irrational expectations and further influence residents’ consumption, and conduct an empirical test on this hypothesis by making use of the econometric model.

## Literature review

Early consumer theories were established based on certain conditions. In The General Theory of Employment, Interest, and Money, Keynes (1936) put forward the absolute income hypothesis, which believed in a stable functional relationship between current consumption and current income, i.e., the level of consumption was determined by the absolute income and marginal propensity to consume decreases progressively. Duesenberry (1949) held that consumption depended on people’s relative income instead of current absolute income, and consumption showed demonstration effect, irreversibility, ratchet effect and recovery effect. According to the permanent income hypothesis of Friedman (1956), people made consumption plans based on their permanent income, i.e., income they could obtain for a long time, but not their short-term income. Modigliani (1955) raised the life cycle hypothesis, which thought that everyone arranged consumption according to the expected income in his/her whole life, saved money when young and used savings when old, to achieve smooth consumption in his/her whole life. These hypotheses had stable and good expectations for future income and believed that consumers had certain expectations for the future; however, as a matter of fact, future income is uncertain. Only when uncertainties have been taken into consideration can we truly explain consumer behaviors.

Being an objective phenomenon in the development of human society, uncertainties exert influence on social and economic development and human life behaviors all the time. Frank (1921) defined an uncertainty as an unforeseen and unmeasurable change. It was Leland who first incorporated uncertain factors in the consumption model. He believed that after uncertainties had been introduced, consumers’ savings not only enabled the equal distribution of wealth throughout the life cycle, but also prepared consumers for any uncertain incident as precautionary savings. Hall and Mishkin (1982) proposed the Random Walking Hypothesis by introducing the rational expectation method into the life cycle and permanent income hypothesis. This hypothesis considered that consumers’ consumption trajectory was a course of random walking by which consumers sought maximum utility based on rational expectations (Hall and Mishkin, 1982). During the demonstration of the random walking hypothesis, Flavin (1997) advanced the Excess Sensitivity theory of consumption, which discovered the significant positive correlation between consumption and foreseen labor income (Flavin, 1997). Campbell and Deaton (1989) raised the Excess Smoothness of consumption, pointing out the great difference between actual consumption changes and theoretical estimates (Deaton, 1991). According to the liquidity constraint theory put forward by Deaton, Zeldes, et al., consumers were subject to constraints as they could not obtain loans from financial or non-financial institutions and individuals to satisfy their consumption needs in case of any uncertainty. Deaton (1991) (Carroll, 1994) and Carroll (1994) proposed the buffer stock savings hypothesis by combining the precautionary savings hypothesis and liquidity constraint theory, which considered savings as a kind of buffer stock that aimed to maintain normal consumption in a bad situation or increase consumption in a good situation (Dynan, 1993).

In the 21st century, scholars have conducted many residents’ consumer behaviors under the uncertain environment. Johan (2001) studied on the impact of preventive savings on Swedish consumption (Johan, 2001). Scotti (2016) using surprise and uncertainty indexes to study on the real-time aggregation of real-activity macro surprises (Scotti, 2016). Gangling Cao, Guangsheng Wan. (2016) Using the survey data from 26 provinces and the special survey data of migrant workers’ household consumption in Shanghai, this paper makes an exploratory study on the changes in the consumption structure of migrant workers’ families at different stages of their life cycle. The results show that the traditional family life cycle model cannot show the different changes in the consumption structure of migrant workers’ families at different stages of their life cycle (Gangling and Guangsheng, 2016). Liu and Zhang (2018) researched on residents’ consumer behaviors between 1978 and 2012. The author measured the uncertainty of expected income and consumption expenditure using deviation adjustment rate and conditional variance, and found that uncertainties would impede the improvement of residents’ consumption. Targeting the “spillover” of domestic consumer demand, (Liu and Zhang, 2018). Park J S, Suh D. (2019) discussed uncertainty and household portfolio choice using evidence from South Korea. Bahmani-Oskooee and Maki Nayeri (2020) using the data of G7 countries, discusses the relationship between policy uncertainty and consumption in the case of policy asymmetry (Park and Suh, 2019). Wei and Yang (2017) focused on residents’ consumption upgrading, established the theoretical analysis framework “income structure – social security – residents’ consumption,” revealed the basic features of Chinese residents’ consumption between 2003 and 2014 according to the elastic calculation results of income in the ELES model, and tested the theoretical framework by making use of the dynamic regression panel data model. With the outbreak of COVID-19, The impact of the epidemic on consumer has gradually deepened, scholars have discussed the impact of the COVID-19 epidemic on consumers from various perspectives. Omar Nor et al. (2021) discusses the panic buying behavior of consumers during the COVID-19: research the impact of uncertainty, severity cognition, scarcity cognition and anxiety (Omar Nor et al., 2021).

To sum up, economists have discussed the changes in residents’ consumer behavior decisions in the context of uncertainties from different perspectives, and believed that consumer behaviors would be subject to the influence of uncertain factors in the future and become irrational. Hence, further studies shall be conducted on how the uncertain expectations affect residents’ consumer behavior, and, especially considering the impact of the current pandemic, on how to adopt policy measures to reduce residents’ uncertain feelings and promote rural residents’ consumption, thus to boost economic growth (Omar Nor et al., 2021). Therefore, this paper will discuss how rural residents’ irrational expectations that result from uncertainties affect residents’ consumption decisions, conduct an empirical test on the theory based on the econometric model, and finally propose policy suggestions (Mankiw and Stephen, 1991).

## Hypothesis and proposition

### Causes of Chinese rural residents’ uncertainty about consumption

Along with the rapid development of China’s economy and continuous urbanization since the Reform and Opening Up, rural residents embrace increasing employment opportunities and rapidly growing income. From 2002 to 2021, Chinese rural residents’ *per capita* disposable income had increased from CNY 2,529 to 18,931 by 6.49 times. During this period, residents had certain expectations for income growth in the future, and believed that income would increase over time. However, seeing the continuation of the pandemic, geopolitical conflict and the general recession of global economy, residents are feeling more uncertain about the future, while these uncertain feelings aggravate the uncertainties of income, expenditure and price (XiaoHua et al., 2016).

### Rural residents’ income Is uncertain

1. Rural residents’ wage income is uncertain.

Along with the economic development and accelerated urbanization in China in recent years, more urban jobs are provided to peasants. An increasing number of peasants work in cities, forming the new group of migrant workers. Wage income has become the major source of income of rural residents. However, migrant workers’ income is uncertain. Migrant workers cannot enjoy the welfare and social security system for urban residents due to the dual household registration system in China; the income and benefits of migrant workers from urban jobs are unstable; and they may also suffer from wage arrears due to their average cultural quality and less stable jobs. Therefore, compared with urban residents, migrant workers’ wage income is more uncertain (Longfei and Jun, 2019).

2. Production and operating income is vulnerable to climate changes and market risks, and is therefore uncertain.

The uncertainties of production and operating income mainly come from natural risks and market risks of agriculture. First, as agricultural producers, Chinese rural residents lack the ability to resist natural disasters. Bahmani-Oskooee and Maki Nayeri (2020) Since agriculture is significantly influenced by the climate, rural residents’ income is extremely unstable, with their income increasing in a good year and decreasing in a bad year.

**Figure 1:**
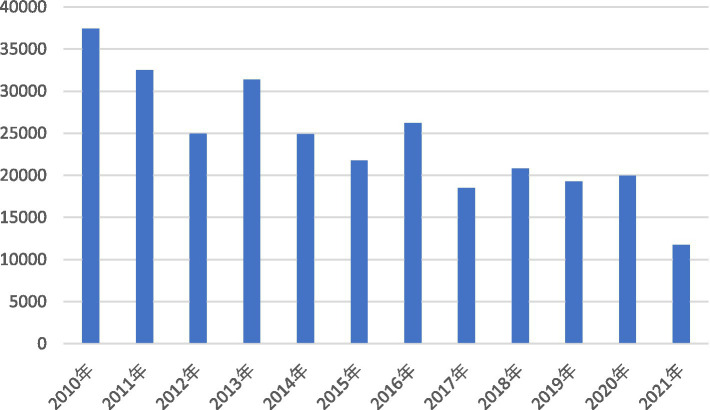
Change in rural disaster-stricken area in china between 2010 and 2021.

Generally, agriculture is seriously affected by natural disasters. From 2010 to 2021, the rural disaster-stricken area ranged between 10,000 and 40,000 thousand hectares in China, which was above 20,000 thousand hectares before 2016, and gradually decreased in recent years until 11,739 thousand hectares in 2021 (Figure 1).

**Figure 2:**
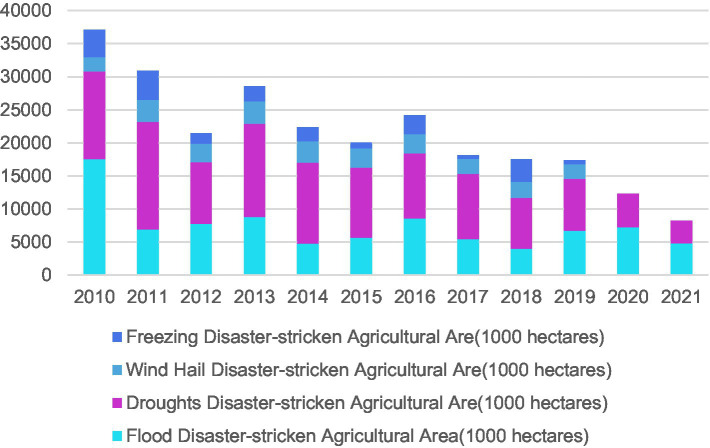
Composition of disaster-stricken agricultural area in China.

China’s agriculture suffers from such natural disasters as floods, droughts, wind hail, and freezing, among which floods and droughts make the major natural disasters faced by Chinese rural areas that hit a large area, jointly occupying 85% of the total disaster-stricken area. Wind hail and freezing affect a smaller area, and produce only 15% of the total disaster-stricken area. As a result of frequent occurrence of agricultural natural disasters, residents’ current income has been lowered, and peasants’ income becomes uncertain (Figure 2).

3. Rural residents also encounter many market risks as operators. Agriculture in a market economy is characterized by the low elasticity of the demand price of agricultural products in both the short and long run, as well as the low elasticity of the supply price in the short run but relatively high elasticity in the long run (Dvornak and Kohler, 2007). Therefore, the supply elasticity of agricultural products is greater than the demand elasticity, so agricultural products are always in the buyer’s market, and rural residents have no bargaining power during the supply and sales of agricultural products.
4. Transfer income is temporary income that cannot relieve consumers’ uncertain feelings.

In recent years, rural residents’ transfer income has grown at an accelerated speed. Transfer income mainly refers to various transfer payments from the government, government-sponsored institutions and social groups to households and the income transfer among households, including subsidies such as family planning subsidies, subsistence allowances, allowances for poverty-stricken people, endowment insurance and ecological compensation; the pension, social relief and assistance, disaster-relief funds, regular donations and compensations transferred from the government, non-administrative institutions and social groups to rural households; as well as alimony income, regular donations and compensations, and the income from non-resident members registered in rural areas but working outside (Easaw et al., 2005). However, transfer income is temporary income. According to Milton Friedman’s permanent income hypothesis, consumers’ income is divided into permanent income and temporary income; and permanent income is the regular and stable income, while temporary income is unexpected and unstable income. What decides people’s consumption expenditure is their permanent and long-term income instead of their short-term disposable income. Transfer income exerts no influence on residents’ income expectation since it is not permanent income.

### Rural residents’ consumption expenditure is uncertain

As rural residents are producers and consumers at the same time, their consumption is divided into two parts, that is the productive consumption to purchase the means of production and living consumption to buy the means of subsistence. These two kinds of consumption show a trade-off correlation given the fixed residents’ income, and often mix together. When the price of the means of agricultural production increases, productive consumption has the crowding-out effect on living consumption (Nadenichek, 2007).

From the perspective of living consumption, various expenditures that were originally borne by enterprises or the government become real expenditures of individuals along with the reform of housing, education and medical system, and such expenditures are subject to numerous uncertainties over time and severely affect residents’ consumption. In recent years, the health expenditures of rural residents have grown rapidly from *per capita* CNY 107 in 2002 to 1,580 in 2021 by 13.76 times. The main reason lies in the imperfect rural social security system by which residents have to spend a lot on illness treatment. The *per capita* health expenditure in rural areas is higher than that of urban residents, mainly due to the single form, narrow coverage, low socialization, weak supportability and unstable fund sources of rural social security system as well as the imperfect security law. Residential consumption expenditure has grown at a high speed in recent years, from *per capita* CNY 326 in 2002 to 3,315 in 2021, and the proportion of residential consumption in *per capita* consumption has fluctuated around 20%. Housing is a very important means of production and subsistence for rural residents, who have rigid demand on houses. To increase income, rural residents may build house of the demand and increase the housing expenditure, resulting in the reduction of other consumption expenditures.

### Rural residents’ phased consumption derived from traditional culture exerts significant influence on rural residents’ uncertain feelings

According to the life cycle hypothesis, rational consumers with certain expectations will arrange for their lifetime consumption based on their lifetime labor income and property income, with the hope of achieving stable consumption in every period of life, to make their lifetime consumption expenditure equal to the sum of their labor income and property income (Segal et al., 2015). Chinese rural residents are exposed to phased consumption rather than smooth consumption. For upcoming important events, consumers will make precautionary savings several years in advance to cope with uncertain events. Such important events include marriage, having a baby, child’s education, elderly care and any major disease that may occur at any time. They will make savings for a period of time before these events, when their consumption is less than their income; and when these big events come, they will use the savings for consumption, so in this period, consumption overtakes income (Baker et al., 2016). Therefore, rural residents often have to make a choice between consumption and savings with their current income, which constitute phased consumption showing a trade-off correlation. In the light of the above hypothesis, consumption arrangements of Chinese rural residents can be divided into 10 phases, including five phases of savings and five of consumption, with each varying in the length of time due to different consumption goals. These 10 phases are, respectively, savings before marriage, consumption for marriage, savings for consumer staples, purchase and construction of fixed assets, savings for children education, consumption for children education, savings for children marriage, consumption for children marriage, savings for elderly and medical care, and consumption for elderly care. It can be seen than rural residents’ whole life can be divided into different phases with different characteristics (Dimab et al., 2017).

To sum up, rural residents in China are faced with greater uncertainties resulting from uncertain income and expenditure and consumption habits. In this case, rural residents have irrational expectations for future income and expenditure rather than rational expectations described by Western scholars (Guojin et al., 2018). As future expenditure is not yet certain, consumers lack necessary information on external environment, particularly the future economic information that can hardly be obtained, and the world is exposed to continuous pandemic, geopolitical conflicts, severe economic regression and the domination of de-globalization thoughts, rational economic subjects attempt to expand their “vision” to the far future and achieve utility maximization. Under these circumstances, consumers will necessarily sacrifice consumption for their feelings of certainty about the future, which will be reflected by low consumption, increase of precautionary savings and residents’ phased consumption.

### Hypothesis

The uncertainty of external environment gives rise to consumers’ uncertain expectations, and consumers’ such irrational expectations on future income, market and price will further influence residents’ consumer decisions. In order to completely explain rural residents’ consumption in this specific historical period, the author here puts forward the basic hypotheses on Chinese rural residents’ consumer behaviors based on Western scholars’ research method (Shin and Kim, 2018).

*Hypothesis 1*: Since consumers obtain independent income, they start to spend their income and wealth in different phases of their life cycle to achieve maximum utility of each phase according to their expected income as rational consumers; but in case of uncertain economic life, consumers will balance consumption and savings to improve their resistance to risks.

*Hypothesis 2*: Faced with both budget constraints and liquidity constraints, consumers’ consumption is divided into two parts, productive consumption and living consumption. In general, consumers always value productive consumption over living consumption.

*Hypothesis 3*: Households adjust their expense to their income. As affected by inherent traditional culture and consumption habits, rural residents never borrow money. It is a traditional virtue of the Chinese nation to have neither internal debts nor external debts. While urban residents have got used to “overdraft consumption,” most rural residents are still subject to the “closed budget constraint.”

*Hypothesis 4*: To meet necessary consumption expenditures including marriage, housing, children education, children marriage, medical care and elderly care, most households are economizing on expenditure in their daily consumption, and save some money to cope with consumption peaks. Hence, residents’ consumption is periodical, shown as the repeated cycle of savings – consumption – savings – consumption.

*Hypothesis 5*: In rural areas with the imperfect social security system, residents’ consumption is closely related to their phases of life, and associated with specific events, including marriage, housing, children education, medical care, elderly care, etc. They have to make a choice between consumption and savings for the sake of these necessary costs, so consumers’ consumption and income show a trade-off correlation in different phases.

*Hypothesis 6*: Characterized by additivity, consumers’ utility function presents the principle of desirability, which means the consumption of more goods (labor) always overtakes the consumption of less goods (labor), but the marginal utility diminishes along with the increase of consumption quantities.

Based on above hypotheses, consumer behaviors of consumers with uncertain expectations will be more cautious. The uncertainty affects consumers’ behaviors in two ways. First, it leads to residents’ cautious consumption, mainly embodied in the lower consumption expenditure. Second, it shakes residents’ confidence and results in the increase of precautionary savings. With fixed income, consumers will make more savings and spend less in the case of greater uncertainties (Morikawa, 2019). On the contrary, the weaker feelings of uncertainties will motivate consumers to spend more and save less. Considering the continuous pandemic, consumers will feel more uncertain, which will further influence their consumption decisions.

## Model selection, index design and data source

### Model selection

According to Franco Modigliani’s life cycle hypothesis, everyone arranges consumption according to the expected income in his/her whole life, saves money when young and uses savings when old, to achieve smooth consumption in his/her whole life. The consumption and saving decision of each household in certain time reflects the household’s efforts to achieve the ideal distribution of consumption in their life cycle, while each household’s consumption is subject to their c cycle, which can be expressed as:


C=a·WR+c·YL

In this formula, WR stands for the actual wealth, and a means the marginal propensity to consume wealth, i.e., the proportion of the wealth consumed each year; meanwhile, YL refers to labor income, and c means the marginal propensity to consume labor income, i.e., the proportion of labor income consumed each year.

Individual consumers with uncertain expectations will not arrange their income and consumption based on their life cycle, but become more myopic. The consumption and income of each phase and the savings at the end of the phase are denoted by *c_i_*, *y_i_*, and *s_i_*, where i = 1, 2, …, 10; the initial wealth of individual consumers is *W*_0_; the risk-free interest rate (savings rate) is *r_f_*; and the discount rate is denoted by *r*. Individual consumers will maximize the utility in each phase, i.e.:


$$\max U=U\left(c_{1},c_{2},\Lambda ,c_{10}\right)$$

$$\text{Meet:}\;\overset{10}{\underset{i=1}{\sum }}\frac{1}{{\left(1+r\right)}^{i}}c_{i}+\overset{10}{\underset{i=1}{\sum }}\frac{1}{{\left(1+r_{f}\right)}^{i}}s_{i}=\overset{10}{\underset{i=1}{\sum }}\frac{1}{{\left(1+r\right)}^{i}}y_{i}=W_{0}$$

According to the Lagrange multiplier method, consumers’ optimum consumption path is to follow the plan:


$$\max_{c_{i},\lambda }L=U\left(c_{1},c_{2},\Lambda ,c_{10}\right)$$

$$+\lambda \left(\overset{10}{\underset{i=1}{\sum }}\frac{1}{{\left(1+r\right)}^{i}}c_{i}+\overset{10}{\underset{i=1}{\sum }}\frac{1}{{\left(1+r_{f}\right)}^{i}}s_{i}-\overset{10}{\underset{i=1}{\sum }}\frac{1}{{\left(1+r\right)}^{i}}y_{i}=W_{0}\right)$$

Upon uncertain conditions, individual consumers have to maximize their utility by accomplishing phased goals in the case of liquidity constraints. In this case, the current consumption will be less than the income, as they will save money for the next phase when consumption will overtake income. Therefore, each two phases constitute a whole, and the life cycle of each individual can be considered as a fluctuated course of consumption with two phases as a feature. Society is a whole made up of individuals, so consumption of society also follows such phased consumption model. In the meantime, each phase shows little relation with other phases (except the adjacent phases), and the savings of the previous phase can be considered as the initial wealth of the current phase (Xuheng and Xin, 2018). Hence, for the convenience of analysis, individual consumers’ optimum consumption can be converted into two discrete phases, that is the current phase and the future phase. Hence, the optimum course of consumption should be:


$$\max_{c_{1},c_{2}}U=U_{1}+\beta U_{2}$$

Meet budget constraints: $(c_{1}+s_{1}=W_{0}\left(1+r_{f}\right)+y_{1}$


$$c_{2}+s_{2}=s_{1}\left(1+r_{f}\right)+y_{2}$$

Since individual consumption utility is a function of actually obtained consumer goods, the unity function can be expressed as:


$$U_{i}=U_{i}\;\left(c_{i},\frac{p_{i}}{p_{i-1}}\right),\;\text{where:}\;\frac{\partial U_{i}}{\partial c_{i}}>0,\frac{\partial U_{i}}{\partial \left(p_{i}/p_{i-1}\right)}<0,\;i=1,2.$$

Eliminate the intermediate parameter *s_1_* to convert the above two budget constraint conditions into one constraint condition comprising the two phases:


$$c_{1}+\frac{1}{\left(1+r\right)}c_{2}+\frac{1}{\left(1+r\right)}s_{2}=y_{1}+W_{0}\left(1+r_{f}\right)+\frac{1}{\left(1+r\right)}\;y_{2}$$

In the meantime, according to the properties of the utility function, if the utility monotonously increases with consumption, any utility function with the same nature as consumption can express such relationship. Considering the relationship between real consumption data and other factors, the hypothetical utility function should be expressed as:


$$U_{i}={\left(\frac{p_{i}}{p_{0}}\right)}^{-1}\log c_{i},i=1,2.$$

Based on the Lagrangian first-order condition for optimum consumption, the general form of the consumption function can be obtained as follows:


$$c_{i}=c_{i}\;\left(\frac{p_{0}}{p_{1}},y_{1},\frac{1}{\left(1+r\right)},r_{f},W_{0},s_{2}\right)\text{,}\;i=1,2.$$

It should be noted that the analysis we conduct now only targets individual consumption behaviors from a microscopic perspective. If the utility functions for all residents and the constraint conditions they meet are consistent, the consumption function of all residents could be the simple magnification of individual economic behaviors; in other words, the whole society presents fluctuated consumption (Peter, 2019). Taking this into consideration, the consumption function of Chinese rural consumers, especially rural residents based on China’s national conditions can also be expressed as:


$$C=C\left(P,P_{0},Y,\frac{1}{\left(1+r\right)},r_{f},W_{0},S\right)$$

The annual data for empirical test come from China Statistical Yearbook 1978–2006, and the sample period is 1978–2006. We put forward the following econometric model based on the consumption functions above:


$$c_{t}=\alpha_{0}\overset{p}{\underset{i=0}{\sum }}\alpha_{i}y_{t-i}+\alpha_{p}P_{t+1}+\alpha_{i}i_{t}\overset{q}{\underset{j=0}{\sum }}\alpha_{j}s_{t-i}+\varepsilon_{t}$$

where $P_{t+1}$ refers to residents’ expected inflation rate of the next phase.

### Index design

Dependent variable: *per capita* consumption expenditure of rural residents. Consumption is the behavior by which residents spend money to maintain their daily life. When consumers hold certain expectations, a stable relationship is seen between rural residents’ *per capita* expenditure and residents’ income. This index is obtained by taking the log of the rural residents’ *per capita* consumption expenditure deducting rural residents’ consumption price index (100 for the previous year) pursuant to China Statistical Yearbook.

Independent variable: We selected rural residents’ *per capita* disposable income, expected inflation rate, interest rate and household savings as independent variables. Being an internationally accepted statistical indicator of household income, *per capita* disposable income reflects the influence of income level and income distribution gap on consumption expenditure (Mingyue and Xuheng, 2020). Rural residents’ *per capita* disposable income means the income they can freely use, which is calculated by deducting individual income tax and non-tax payments from residents’ actual income. The expected inflation rate is an index to measure people’s psychological expectations of the inflation rate, which is used to measure residents’ expectations of future uncertainty; Interest rate, considering that the main influencing factor of liquidity constraint is interest rate, interest rate is selected as one of the uncertain factors of future liquidity constraint (Xiaojuan and Lijun, 2021). This paper is expressed by the one-year deposit interest rate stipulated by the People’s Bank of China. If the interest rate of a certain year is adjusted frequently, the interest rate level of that year is calculated based on the term weighting of each interest rate level. Saving is an important factor affecting the current income of residents’ consumption, In the case of a certain income, residents’ savings and consumption have the characteristics that one is rising, the other is falling. Therefore, we take the amount of savings deposit as a variable to examine the impact of household savings on consumption.

All data in this paper come from China Statistical Yearbook, with data between 2002 and 2022 as the sample. For Chinese urban residents’ *per capita* consumption expenditure and *per capita* disposable income and savings deposit, the actual values are obtained by means of price deflator to exclude the effect of price.

## Empirical test results and evaluation

**Figure 3:**
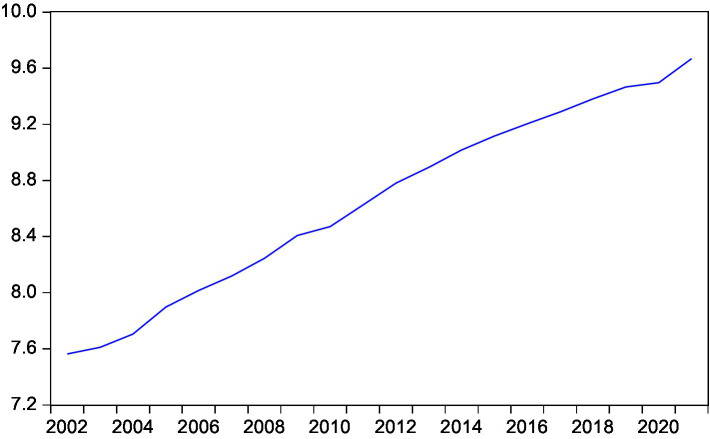
Trend of rural residents’ actual consumption.

**Figure 4:**
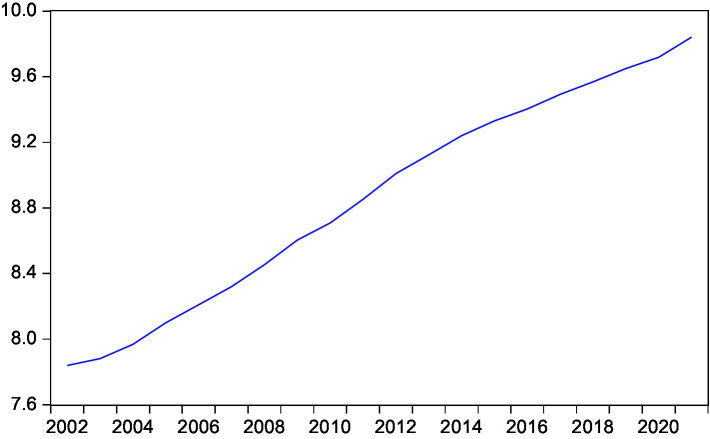
Trend of rural residents’ actual income.

**Figure 5:**
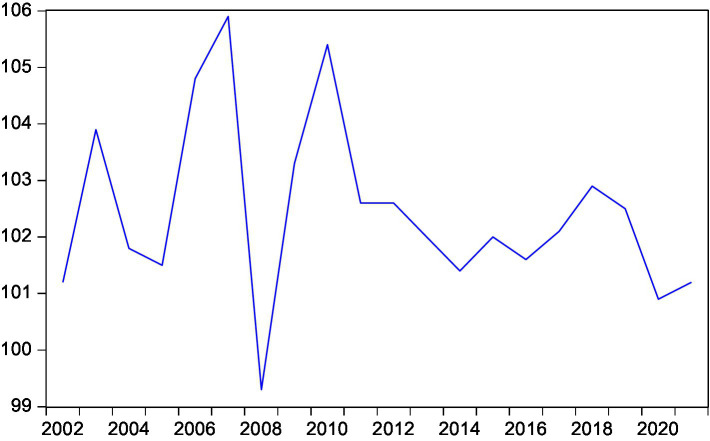
Trend of expected inflation rate.

**Figure 6:**
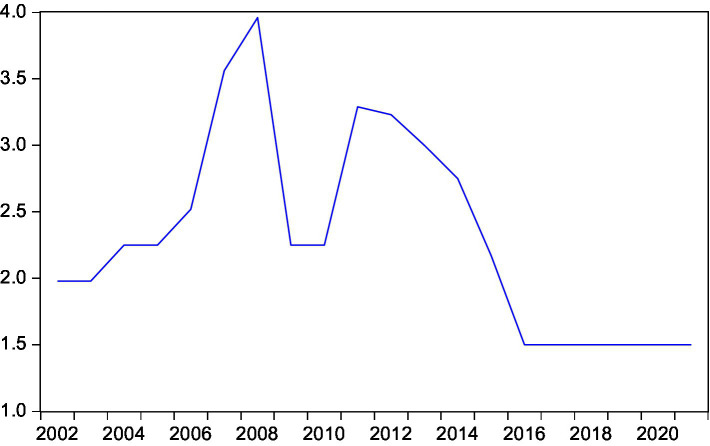
Trend of interest rate.

**Figure 7:**
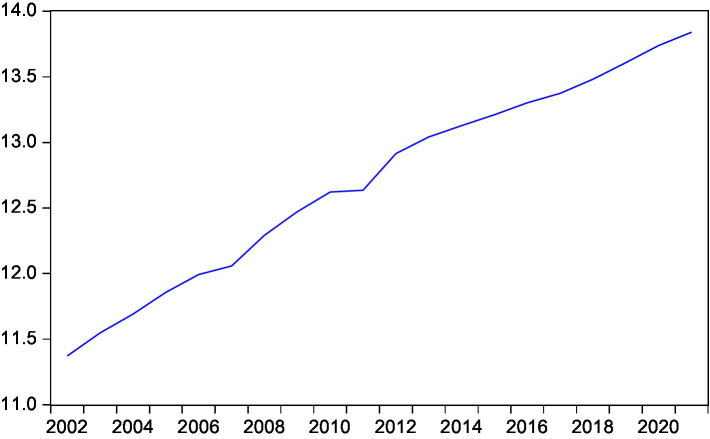
Trend of national residents’ savings.

This paper intends to conduct an empirical test on the long and short-term conduction effect between rural residents’ consumption and rural residents’ disposable income, expected inflation, interest rate and national household savings by means of the cointegration test and the error correction model (Figures 3–7).

### Unit root test

With the rural residents’ consumption and rural residents’ disposable income, expected inflation, interest rate and national household savings in China between 2002 and 2021, this paper conducts an empirical test on the long and short-term conduction effect between rural residents’ consumption and rural residents’ disposable income, expected inflation, interest rate and national household savings by means of the cointegration test, the ECM model and Granger causality test. The logarithms of the five values above are used as the variables of the model, respectively denoted as $Ln\left(c\right),Ln\left(y\right)$, $p_{t+1}$, r and $Ln\left(s\right)$, where the added value of each sector represents the composition of economic growth.

### Unit root test on $Ln\left(c\right)$

Conduct ADF test on the $Ln\left(c\right)$ series, determine the optimum lag order (*p* = 1) based on the SC rule, and obtain the test results (including the trend and constant) as shown in Table 1.

**Table 1:** Ln(c) ADF Test Results.

Difference order	t-statistic	Critical value (5%)	Prob.*
0	−1.322222	−3.6736	0.8501
1	−4.369455	−3.0404	0.0035**

*** Means being significant at the 1% level.

** Means being significant at the 5% level.

* Means being significant at the 10% level.

Seen from Row 1 of Table 1, at the significance level of 1%, t = −1.322222 > −3.6736, demonstrating that the original hypothesis is accepted and $Ln\left(c\right)$ is non-stationary. The results of the ADF test on $\Delta Ln\left(c\right)$ is displayed in Row 2, suggesting that the original hypothesis is rejected at the 5% significance level of $\Delta Ln\left(c\right)$ and $\Delta Ln\left(c\right)$ is considered as stationary series. In other words, $Ln\left(c\right)$ is an integrated series of order one, denoted as $Ln\left(c\right)\sim I\left(1\right)$.

### Unit root test on $Ln\left(y\right)$

Conduct ADF test, determine the optimum lag order based on the SC rule, and obtain the test results (including the trend and constant) as shown in Table 2.

**Table 2:** Ln(y) ADF Test Results.

Difference Order	t-statistic	Critical Value (5%)	Prob.*
0	−0.736989	−3.0299	0.9933
1	−3.477634	−3.2869	0.007*

*** Means being significant at the 1% level.

** Means being significant at the 5% level.

* Means being significant at the 10% level.

According to the results of ADF test, $Ln\left(y\right)$ accepts the original hypothesis as it is non-stationary; and $\Delta Ln\left(y\right)$ rejects the original hypothesis as it is stationary. The same conclusion can be obtained through other test methods that $Ln\left(y\right)$ is an integrated series of order one, denoted as $Ln\left(y\right)\sim I\left(1\right)$.

### Unit root test on expected inflation rate $p_{t+1}$

Conduct ADF test, determine the optimum lag order based on the SC rule, and obtain the test results (including the constant at the zero-order differential, and including the trend and constant at the first-order differential) as shown in Table 3.

**Table 3:** pt+1 ADF Test Results.

Difference order	t-statistic	Critical value (5%)	Prob.*
0	−0.209267	−1.9628	0.5960
1	−7.666062	−3.0522	0.0000**

*** Means being significant at the 1% level.

** Means being significant at the 5% level.

* Means being significant at the 10% level.

As shown in Table 3, the *p* value of the expected inflation rate $p_{t+1}$ in the ADF test is 0.5960, demonstrating that the unit root is found at 59.60% and $p_{t+1}$ is non-stationary; and $\Delta p_{t+1}$ is non-stationary and rejects the original hypothesis. The same conclusion can be obtained through other test methods that $p_{t+1}$ is an integrated series of order one, denoted as $p_{t+1}\sim I\left(1\right)$.

### Unit root test on interest rate r

Conduct ADF test, determine the optimum lag order based on the SC rule, and obtain the test results (including the trend constant at the zero-order differential, and excluding the trend and constant at the first-order differential) as shown in Table 4.

**Table 4:** r ADF Test Results.

Difference order	t-statistic	Critical value (5%)	Prob.*
0	−1.562555	−3.0207	0.4814
1	−4.956881	−1.9614	0.0001***

*** Means being significant at the 1% level.

** Means being significant at the 5% level.

* Means being significant at the 10% level.

According to the ADF test results in Table 4, the interest rate r accepts the original hypothesis and is non-stationary; and Δr is non-stationary and rejects the original hypothesis. The same conclusion can be drawn through other test methods that r is an integrated series of order one, denoted as $r\sim I\left(1\right)$.

### Unit root test on $Ln\left(s\right)$

Conduct ADF test, determine the optimum lag order based on the SC rule, and obtain the test results (including the trend constant at the zero-order differential, and excluding the trend and constant at the first-order differential) as shown in Table 5.

**Table 5:** Ln(s) ADF Test Results.

Difference order	t-statistic	Critical value (5%)	Prob.*
0	−1.732646	−3.6736	0.6962
1	−4.868837	−3.0404	0.0013***

*** Means being significant at the 1% level.

** Means being significant at the 5% level.

* Means being significant at the 10% level.

According to the ADF test results in Table 5, the $Ln\left(s\right)$accepts the original hypothesis and is non-stationary; and $\Delta Ln\left(s\right)$ is non-stationary and rejects the original hypothesis. The same conclusion can be drawn through other test methods that $Ln\left(s\right)$ is an integrated series of order one, denoted as$Ln\left(s\right)\sim I\left(1\right)$.

To sum up, $Ln\left(c\right),Ln\left(y\right)$, $p_{t+1}$, r and $Ln\left(s\right)$ are all integrated series of order one, on which we can conduct the cointegration test.

### Cointegration test

(1) As $Ln\left(c\right),Ln\left(y\right)$, $p_{t+1}$, r and $Ln\left(s\right)$ are all integrated series of order one, we can conduct the cointegration test based on Engle-Granger two-step method on these five variables, and establish the model below:


$$c_{t}=\alpha_{0}+\alpha_{y}y_{t}+\alpha_{p}p_{t+1}+\alpha_{i}r_{t}+\alpha_{s}s_{t}+\varepsilon_{t}$$

Then we adopt OLS estimation, conduct the unit root test on the residual term ${\hat{e}}_{t}$, and determine the lag order based on the SC rule. The results are shown in Table 6.

**Table 6:** Residuals of the unit root test Results.

Variable	Inspection type	ADF	Prob.*
${\hat{e}}_{t}$	None	−4.402389***	0.0416**
	Intercept	−3.872457**	0.0042***
	Trend and intercept	−4.402389***	0.0003***

${\hat{e}}_{t}$means residual serial.

*** Means being significant at the 1% level.

** Means being significant at the 5% level.

* Means being significant at the 10% level.

The results show that ADF test statistics is-3.872457, P is 0.0416, and the critical value of EG two-step method is greater than t statistics, so it rejects H0 and suggests that the residual serial is stationary without unit root, and cointegration relationship is seen among these variables. Therefore, regression estimation can be performed.


$$\begin{array}{l} Ln\left(c_{t}\right)=-0.98+0.92Ln\left(y\right)+0.003p_{t+1}-0.002r\\ \;-0.095793Ln\left(s\right)+{\hat{e}}_{t},t=1,2,\cdots T \end{array}$$

According to the estimates of the model, rural residents’ consumption shows positive correlation with current income and expected inflation rate and negative correlation with the interest rate and savings. The results display a long-term balance among these variables.

### Establishment of the error correction model

These variables are balanced in the long run, but may be out of balance in the short term, so the ECM model is adopted to analyze the dynamic relations among these variables. Establish the ECM model based on $ecm_{t}={\hat{e}}_{t}$ with the residual serial ${\hat{e}}_{t}$ as the error correction term, and obtain the short-term dynamic disequilibrium equation by means of regression:


$$\begin{array}{l} \Delta Ln\left(c_{t}\right)=-0.155825+0.837421\Delta Ln\left(y_{t}\right)-0.000987\Delta p_{t+1}\\ \;-0.009057\Delta r_{t}-0.050693\Delta lns+0.938302{\hat{e}}_{t-1} \end{array}$$

$$\begin{array}{l} t=\left(-5.473186\right)\;\left(9.257759\right)\;\left(-0.589445\right)\left(-1.313997\right)\\ \;\left(-0.806073\right)\left(5.817869\right) \end{array}$$

$$R^{2}=0.912\;F=26.99$$

In the error correction model above, the difference reflects the impact of short-term fluctuation. The short-term changes in rural residents’ *per capita* consumption in China are divided into two parts, respectively the impact of short-term fluctuation of rural residents’ *per capita* disposable income, and the impact of deviation from long-term equilibrium. The error correction term reflects the adjustment effort against the deviation from long-term equilibrium. As indicated by the factor estimate (0.938302), when the short-term fluctuation deviates from long-term equilibrium, the adjustment effort of (0.938302) will be applied to return to the state of equilibrium.

## Conclusion and policy suggestions

### Research conclusion

The following conclusions can be drawn upon the analysis of the consumption decisions of Chinese rural residents with uncertain expectations:

Uncertainty is an inevitable and objective reality. Normally, certain economic variables may produce uncertainties, while the uncertain external environment will aggravate residents’ uncertain feelings and thus affect residents’ consumption decisions (Huang and Luk, 2020). Also as a result of uncertainties, Chinese rural residents have irrational expectations, which will not only lead to residents’ cautious consumption but also undermine residents’ confidence. Therefore, instead of arranging consumption based on the whole expected income to smoothen their lifetime consumption, residents make phased plans of consumption since they have independent income, to cope with consumption peaks in different phases. In this case, the irrational expectations resulting from uncertain feelings will lead to rural residents’ short-sighted consumption behaviors. Consumers will “rationally” choose between consumption and savings with their income to accomplish their phased consumption goals and tend to make savings against major events.

Consumers’ income, consumption, consumption habits, liquidity constraints and precautionary savings jointly produce the feelings of uncertainty, which will lead to irrational expectations that induce rural residents’ short-sighted consumption. Consumers will “rationally” choose between consumption and savings with their income to accomplish their phased consumption goals and tend to make savings against major events. Therefore, corresponding policies measures shall be carried out to eliminate the uncertain feelings of rural residents, and ultimately achieve the goal of promoting rural residents’ consumption and economic growth.

### Policy suggestions

The government’s macroeconomic policies are of great significance to rural consumption. Considering the law of the changes in rural consumption, we shall carefully analyze the factors influencing rural residents’ consumption, put forward corresponding policies and measures, and thus enable the beneficent cycle of “promoting rural consumption – driving the supply-side growth – enhancing high-quality economic development.” The following measures shall be included:

### Increase rural residents’ income and achieve consumption and rural revitalization

As a special consumer group, rural residents are consumers and direct producers, operators and investors at the same time (Jessika et al., 2022). Various measures shall be applied to increase peasants’ operating income, wage income, net income from property and net income from transfer, thus to guarantee the stable growth of peasants’ income, including:

First, increase the operating income of residents. The prosperous development of agriculture constitutes the foundation for promoting peasants’ income. The net operating income currently occupies 1/3 of the total income of rural residents. We should support the integration of digital economy into rural technologies, reform and upgrade the industrial chain of agriculture, develop high-quality and efficient agriculture, and improve the supply quality of agricultural products and comprehensive agricultural benefits. Second, the employment channels for peasants shall be expanded to increase rural residents’ wage income. For this end, the government shall increase local jobs of rural residents and the job opportunities for migrant workers, strengthen the skill training and vocational education of residents, improve the financial, tax and industrial support policies for employment, and offer stable work opportunities to migrant workers. Third, the land system reform in rural areas shall be strengthened to increase peasants’ income from properties. Finally, as an important part of the improvement of the redistribution regulation system, peasants’ transfer income shall be increased (Ross, 2022).

### Reform the income distribution system to achieve common prosperity

Since the Reform and Opening Up, the income gap has gradually emerged and been widened. To improve the income distribution system by means of the third distribution, we shall promote common prosperity from two dimensions, the “common prosperity of spiritual life” and “common prosperity of material life” for both urban and rural areas. To achieve this goal, the follows shall be done: ①Cultivate the subjects of social charity. Inspire social capital registered charity institutions to play a more active part in charity activities in a voluntary manner, and especially encourage and lead high-income groups to participate in charities; ② Improve the third distribution mechanism in the rural areas. Establish the emerging third-distribution platforms such as online charity activities and Internet donation platforms; ③ Consummate the donation methods by transforming the money and material donation to equity, skill and technology donation; ④ and improve the system of encouragement for third distribution, and promote the third distribution through fiscal policies, tax policies and industrial policies. We shall give full play to the market mechanism, social mechanism, administrative mechanism, cultural mechanism, digital governance mechanism and common prosperity evaluation mechanism.

### Improve the rural financial system and financially enable rural consumption

Finance makes the foundation of the national economy, while the development of rural finance constitutes the major motive force for the development of rural economy and rural consumption (Yoon et al., 2021). Rural residents’ consumption has been restrained due to the liquidity constraints. ①Strengthen the construction of rural finance infrastructure. Establish new service points in addition to the existing rural finance network, and extend the coverage of ATMs to remote areas. ② Innovate the systems and mechanism of rural financial services. The consumption system plays as an important guarantee for finance-driven rural consumption. ③ Guide financial institutions to allocate financial resources rationally in rural areas, innovate consumption credit products there and enrich the models of credit mortgage and pledge. ④ Support rural finance with digital economy, apply artificial intelligence, big data and other advanced digital technologies to help with the credit rating of agricultural enterprises and rural residents, establish and improve the query system and credit rating system of rural consumption loans, and thus lower the credit risks of commercial banks. ⑤ Enhance the service capability of agricultural insurance, and promote the innovation of agricultural insurance products. Agriculture is naturally vulnerable to both natural risks and market risks, so agricultural insurance shall be developed to diversify risks.

### Reinforce the construction of rural consumption infrastructure

Coordinate urban and rural development, narrow the urban–rural consumption gap, and especially narrow the urban–rural gap in terms of public services and consumption. Since the rural areas in different regions in China vary greatly in natural resources, population and development potential, the key to narrow the urban–rural gap is to balance the public services and consumption infrastructure of rural residents in different regions. Hence, the following measures shall be adopted: ① Motive rural consumption through technology, and encourage new consumption patterns to march into rural areas. ② Establish the logistics system in rural areas and strengthen the construction of modern distribution network there, to effectively lower the distribution cost in rural areas. ③ Strengthen the water and power supply system and road construction in rural areas. ④ Promote the development of digital rural construction. Establish the big data service system for rural areas and agriculture, incorporate the new-generation information technology in agricultural production and operation, and realize smart agriculture. ⑤ Promote the telecommunication service compensation system, and especially support the information and communication infrastructure construction in remote areas. ⑥ Reinforce digital construction in rural social governance, public services, etc. The government and social capital shall cooperate in investment, to inspire social forces to take a part in rural infrastructure construction.

In conclusion, multiple policies shall be applied at the same time to stimulate rural consumption (Anderson et al., 2022). These policies shall be coordinated with each other in terms of the goals and the implementation date, and shall never contradict with each other, thus to maintain the systematic characteristic of rural consumption. Only by taking all things into consideration can the concerted force of policies be formed, and can the effect of various policies be maximized, finally to eliminate uncertain expectations of rural residents, promote rural consumption and drive the national economic development.

## Data availability statement

The original contributions presented in the study are included in the article/supplementary material, further inquiries can be directed to the corresponding author.

## Author contributions

All authors listed have made a substantial, direct, and intellectual contribution to the work and approved it for publication. LH, XL, and SM were involved in building the framework and searching for information during the conception of the article. LH did the main work of writing the paper. The authors of this paper contributed the equal.

## Funding

This article is funded by Ministry of Education humanities social sciences research project of China Research on the Economic Effects and Policy Implications of Rural Residents’ Consumption Upgrading under the Background of Financial Enabling (No.: 21YJA790019); and Guangdong Province Philosophy Social Science Project “Research on the System Design and Impact Mechanism of Financial Support for Technological Innovation in Guangdong Hong Kong Macao Greater Bay Area” (No.: GD20XYJ06) and Beijing Laboratory of National Economic Security Early-warning Engineering, Beijing Jiaotong University.

## Conflict of interest

The authors declare that the research was conducted in the absence of any commercial or financial relationships that could be construed as a potential conflict of interest.

## Publisher’s note

All claims expressed in this article are solely those of the authors and do not necessarily represent those of their affiliated organizations, or those of the publisher, the editors and the reviewers. Any product that may be evaluated in this article, or claim that may be made by its manufacturer, is not guaranteed or endorsed by the publisher.

## References

[ref1] Anderson S. Rayburn W. Sierra J. Murdock K. McGeorge A. (2022). Consumer buying behavior and retailer strategy through a crisis: a futures studies perspective. J. Mark. Theory Pract. 30, 457–475. doi: 10.1080/10696679.2021.1982648

[ref2] Bahmani-Oskooee M. Maki Nayeri M. (2020). Policy uncertainty and consumption in G7 countries: an asymmetry analysis. Int. Econ. 163, 101–113. doi: 10.1016/j.inteco.2020.06.001

[ref3] Baker S. R. Bloom N. Davis S. J.. (2016). Measuring economic policy uncertainty. Q. J. Econ. 131, 1593–1636. doi: 10.1093/qje/qjw024

[ref100] Campbell J. Deaton A. (1989). Why is Consumption so Smooth? Rev. Econ. Study Rev. Econ. Studies 56, 357–373.

[ref4] Carroll C. (1994). How does future income affect current consumption? Q. J. Econ. 35, 111–148.

[ref5] Deaton A. (1991). Saving and Iiquidity constraints. Econometrica 59, 1221–1142. doi: 10.2307/2938366

[ref6] Dimab B. Dinca M. S. Diam S. M. Dinca G. (2017). Does economic policies uncertainty affect economic activity? Evidences from the United States of America. J. Econ. For. 20, 60–74.

[ref7] Dvornak N. Kohler M. (2007). Housing wealth, stock market wealth and consumption: a panel in review level analysis for Australia. Econ. Rec. 83, 117–130. doi: 10.1111/j.1475-4932.2007.00388.x

[ref200] Duesenberry J. S. (1949). Income, Saving and the Theory of Consumer Behavior, Cambridge.

[ref8] Dynan K. E. (1993). How prudent are consumers. J. Polit. Econ. 101, 1104–1113. doi: 10.1086/261916

[ref9] Easaw J. Z. Garratt D. Heravi S. M. (2005). Does consumer sentiment accurately forecast UK household consumption? Are there any comparisons to be made with the US?, J. Macroecon., 27:517–532. doi: 10.1016/j.jmacro.2004.03.001.

[ref10] Flavin M. A. (1997). The adjustment of consumption to Changjing expectations about future income. J. Polit. Econ. 89, 974–1009.

[ref300] Frank H. K. (1921). Risk, Uncertainty and Profit, Boston and New York Houghton Mifflin Company.

[ref400] Friedman M. (1956). Studies in the Quantity Theory of Money the University of Chicago Press.

[ref11] Gangling C. Guangsheng W. (2016). Family life cycle variation of migrant workers and its impact on their family consumption structure. Manage. World 11, 96–109. doi: 10.19744/j.cnki.11-1235/f.2016.11.008

[ref12] Guojin C. Runze Z. Xiangqin Z. (2018). Economic policy uncertainty and stock risk characteristics. J. Manag. Sci. China 21, 1–27.

[ref13] Hall R. E. Mishkin F. (1982). The sensitivity of consumption to transitory income: estimate from panel data on households. Econometrica 50, 461–481. doi: 10.2307/1912638

[ref14] Huang Y. Luk P. (2020). Measuring economic policy uncertainty in China. China Econ. Rev. 59:101367. doi: 10.1016/j.chieco.2019.101367

[ref15] Jessika M. Vásquez N. Mirza M. C. (2022). Food consumption and food waste behavior in households in the context of the COVID-19 pandemic. Br. Food J. 124, 4477–4495. doi: 10.1108/BFJ-07-2021-0798

[ref16] Johan L. (2001). The effect if precautionary saving on consumption in Sweden. Appl. Econ. 33, 673–681. doi: 10.1080/00036840122493

[ref500] Keynes J. M. (1936). The General Theory of Employment, Interest and Money, London.

[ref17] Liu J. Zhang C. (2018). Study on the impact of uncertainty of expected revenue and expenditure on Residents' consumption behavior -- based on the analysis of urban residents' consumption data in China's economic transition, consumer economy, no.(10): 2015, 31 (05), 10–16+34.

[ref18] Longfei Z. Jun Z. (2019). The evolutionary trend and regional differences of urban household consumption inequality in China. Finance Trade Eco. 5, 143–160.

[ref19] Mankiw N. G. Stephen P. Z. (1991). The consumption of stockholders and nonstock holders. J. Financ. Econ. 29, 97–112. doi: 10.1016/0304-405X(91)90015-C

[ref20] Mingyue S. Xuheng Z. (2020). Heterogeneous consumers, household debt and consumer expenditure. Econ. Perspect. 6, 74–90.

[ref700] Modigliani F. (1955). “Utility Analysis and the Consumption Function: An Interpretation of Cross-Section Data”, Post-Keynesian Economics, ed. By Kenneth K. Kurihara, London.

[ref21] Morikawa M. (2019). Policy uncertainty and saving attitude: evidence from a survey on consumers. J. Consum. Aff. 53, 1297–1311. doi: 10.1111/joca.12230

[ref22] Nadenichek J. (2007). Consumer confidence and economic stagnation in Japan. Jpn. World Econ. 19, 338–346. doi: 10.1016/j.japwor.2006.05.005

[ref23] Omar Nor A. Nazri Muhamad A. Ali Mohd H. Alam Syed S. (2021). The panic buying behavior of consumers during the COVID-19 pandemic: examining the influences of uncertainty, perceptions of severity, perceptions of scarcity, and anxiety. J. Retail. Consum. Serv. 62:102600. doi: 10.1016/j.jretconser.2021.102600

[ref24] Park J. S. Suh D. (2019). Uncertainty and household portfolio choice: evidence from South Korea. Econ. Lett. 180: 21–24. doi: 10.1016/j.econlet.2019.03.009.

[ref25] Peter S. (2019). Uncertain pension income and household saving. Rev. Income Wealth 65, 908–929. doi: 10.1111/roiw.12383

[ref26] Ross Otto A. (2022). Context-dependent choice and evaluation in real-world consumer behavior Louie Kenway scientific reports, vol. 10. 10.1038/s41598-022-22416-5 PMC9588046 36273073

[ref27] Scotti C. (2016). Surprise and uncertainty indexes: real-time aggregation of real-activity macro surprises. J. Monet. Econ. 82, 1–9. doi: 10.1016/j.jmoneco.2016.06.002

[ref28] Segal G. Shaliastovich I. Yaron A. (2015). Good and bad uncertainty: macroeconomic and financial market implications. J. Financ. Econ. 117, 369–397. doi: 10.1016/j.jfineco.2015.05.004

[ref29] Shin S. H., Kim K. T. (2018). Perceived income changes, saving motives, and household savings. J. Financ. Couns. Plan., 29: 396–409. doi: 10.1891/1052-3073.29.2.396.

[ref600] Wei Y. Yang M. (2017). Income Structure, Social Security and Consumption Upgrade of Urban Residents, East China Economic Management, Mar. East China Econ. Manag. 31, 90–99. doi: 10.3969/j.issn.1007-5097.2017.03.012

[ref30] XiaoHua W. Tao W. Jiong Z. (2016). Habit formation, income structure imbalance and the evolution of rural residents' consumption behavior. Econ. Perspect. 10, 39–49.

[ref31] Xiaojuan J. Lijun M. (2021). Internal circulation first, external circulation enabling and higher level double circulation: international experience and Chinese practice. Manage. World 1, 1–18.

[ref32] Xuheng Z. Xin Z. (2018). The analysis of household asset allocation and heterogeneous consumer behavior in China. Econ. Res. J. 3, 21–34.

[ref33] Yoon S. McClean S. Chawla T. Kim J. Koopman J. (2021). Working through an “infodemic”: the impact of COVID-19 news consumption on employee uncertainty and work behaviors, J. Appl. Psychol. 4. doi: 10.1037/APL0000913 34014706

